# Phosphodiesterase Inhibitors as a Therapeutic Approach to Neuroprotection and Repair

**DOI:** 10.3390/ijms18040696

**Published:** 2017-03-24

**Authors:** Eric P. Knott, Mazen Assi, Sudheendra N. R. Rao, Mousumi Ghosh, Damien D. Pearse

**Affiliations:** 1 Herbert Wertheim College of Medicine, Florida International University, Miami, FL 33199, USA; 2 The Miami Project to Cure Paralysis, The Miller School of Medicine at the University of Miami, Miami, FL 33136, USA; m_assi2010@live.com (M.A.); sudhee26@med.miami.edu (S.N.R.R.); mghosh@med.miami.edu (M.G.); DPearse@med.miami.edu (D.D.P.); 3 The Department of Neurological Surgery, The Miller School of Medicine at the University of Miami, Miami, FL 33136, USA; 4 The Neuroscience Program, The Miller School of Medicine at the University of Miami, Miami, FL 33136, USA; 5 The Interdisciplinary Stem Cell Institute, The Miller School of Medicine at the University of Miami, Miami, FL 33136, USA; 6 Bruce Wayne Carter Department of Veterans Affairs Medical Center, Miami, FL 33136, USA

**Keywords:** cyclic nucleotides, CNS, PDE, phosphodiesterase, phosphodiesterase inhibitor, cyclic GMP, cyclic AMP, repair, regeneration, cell death, clinical trials

## Abstract

A wide diversity of perturbations of the central nervous system (CNS) result in structural damage to the neuroarchitecture and cellular defects, which in turn are accompanied by neurological dysfunction and abortive endogenous neurorepair. Altering intracellular signaling pathways involved in inflammation and immune regulation, neural cell death, axon plasticity and remyelination has shown therapeutic benefit in experimental models of neurological disease and trauma. The second messengers, cyclic adenosine monophosphate (cyclic AMP) and cyclic guanosine monophosphate (cyclic GMP), are two such intracellular signaling targets, the elevation of which has produced beneficial cellular effects within a range of CNS pathologies. The only known negative regulators of cyclic nucleotides are a family of enzymes called phosphodiesterases (PDEs) that hydrolyze cyclic nucleotides into adenosine monophosphate (AMP) or guanylate monophosphate (GMP). Herein, we discuss the structure and physiological function as well as the roles PDEs play in pathological processes of the diseased or injured CNS. Further we review the approaches that have been employed therapeutically in experimental paradigms to block PDE expression or activity and in turn elevate cyclic nucleotide levels to mediate neuroprotection or neurorepair as well as discuss both the translational pathway and current limitations in moving new PDE-targeted therapies to the clinic.

## 1. Introduction

According to the Centers for Disease Control and Prevention (CDC), nearly two million people experience a traumatic brain injury (TBI) annually in the United States, resulting in over a quarter of a million hospitalized and more than fifty thousand deceased [1,2,3]. Similarly, there are 15 to 40 new cases per million people (12,000 to 20,000) of spinal cord injury (SCI) that occur annually in the US, with approximately 2.5 million people presently living with SCI worldwide [4]. Currently, the clinical management of neurotrauma is focused on supportive care, minimizing intracranial pressure, hypotension and hemorrhage, while utilizing anti-convulsive therapies and hypothermia to inhibit seizures and prevent neuronal loss in the acute setting. Advancements, however, are being made in moving restorative therapies towards clinical implementation, such as the introduction of cell transplants, which have been demonstrated to promote neurorepair in experimental paradigms [5].

Injury to the CNS produces an instantaneous loss of neuronal and axonal components followed by a series of events termed “secondary injury”. Secondary injury begins acutely after the primary insult and continues for several weeks, expanding the region and severity of tissue injury. Initially, micro-hemorrhages appear in the grey and white matter, followed by interstitial edema. Activation of the coagulation cascade leads to vasospasms and promotes thrombosis, further exacerbating tissue ischemia. At the biochemical level, ionic imbalances, elevated concentrations of pro-inflammatory cytokines and chemokines, and increased levels of glutamate and reactive species all serve to initiate neural cell death [6]. The loss of neural components leads to cyst formation and an encapsulating glial scar, composed of inhibitory chondroitin sulfate proteoglycans, myelin associated inhibitors and other axon growth antagonizing molecules [7,8], which physically and chemically hinder endogenous repair. Furthermore, in contrast to the peripheral nervous system (PNS) in which neurorepair occurs, the regenerative capacity of injured CNS axons is severely restricted in the adult not only by the hostile environment but also by intrinsic differences in neuronal growth programs, reduced expression of regeneration associated genes [7,9] and the absence of supportive cells, such as Schwann cells (SCs), which orchestrate the reparative process in the injured PNS [10,11]. Although a discussion of the involvement of cyclic AMP (adenosine monophosphate) and the use of PDE inhibitors in promoting PNS regeneration involves mention of certain cellular and signaling pathways that are similarly present in the CNS, such an overarching topic extends beyond the scope of the current review. A recent examination of this subject can be found elsewhere [12].

During the last 25 years, at least five pharmacological therapies have been evaluated in Phase III trials for the management of acute injury to the spinal cord. These include Methylprednisolone Sodium Succinate, Naloxone, Tirilazad Mesylate, Nimodipine, and GM-1 (Sygen) [5]. The effectiveness of these agents in providing meaningful improvements in recovery after SCI in humans was either not significant to warrant their broad use clinically or, in the case of Methylprednisolone, led to its use as a recommended treatment acutely in North America to be used within 8 h of injury [13]. A myriad of other experimental neuroprotectants, which have demonstrated efficacy in animal paradigms of TBI or SCI, are being translated to man but have yet to show effectiveness in Phase III clinical trials [14]; these include Riluzole, Minocycline, basic fibroblast growth factor, Cethrin, Anti-Nogo as well as Rolipram. The last of these is a pharmacological agent which antagonizes an enzyme involved in the hydrolysis of cyclic nucleotides, the central topic of the present review.

**Figure 1:**
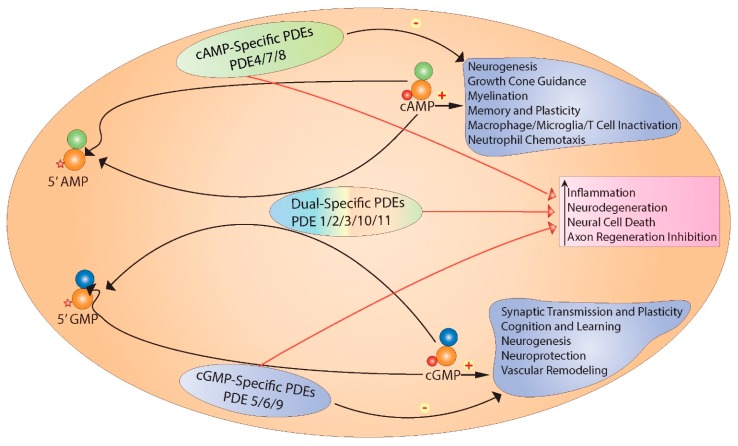
The effects of cyclic nucleotide:PDE signaling on pathological and reparative processes after neurological disease and injury. Cyclic nucleotides and their downstream effectors are critical signaling molecules for a myriad of cellular functions from neurogenesis and myelination to vascular remodeling and neuroplasticity (black arrows). During neurological disease or injury, increased expression and/or activity of various PDE enzymes leads to the hydrolysis of cyclic AMP and GMP to 5′AMP or 5′GMP, respectively (black arrow loops), antagonizing these processes and altering the balance of cell responses towards inflammation, cell death and neurodegeneration (red arrows).

Through the elevation of intracellular cyclic adenosine monophosphate (cyclic AMP) levels, cyclic AMP analogs, adenylyl cyclase (AC) activators, and phosphodiesterase (PDE) inhibitors have been shown to curtail immune cell activation [15,16], antagonize pro-inflammatory cytokine production [17,18,19,20], and promote regeneration after CNS injury [21,22,23] (Figure 1). Furthermore, locomotor behavior was improved with elevated cyclic AMP levels in experimental SCI models [24,25,26]. PDE inhibition as a therapeutic approach to neuroprotection and neurorepair is a promising strategy that aims to restore CNS cyclic AMP levels, which are reduced after neurotrauma [24,27]. Increasing cyclic AMP would then harness the growth and restorative functions of this intracellular signaling molecule. Through cytoskeletal reorganization, cyclic AMP can provide axon guidance toward chemoattractive cues and so direct the regeneration of axons across inhibitory substrates. It also disrupts the secondary injury cycle of neurotoxicity that follows acute CNS injury. Given that intracellular cyclic AMP is degraded solely by PDEs, the introduction of a PDE inhibitor to antagonize cyclic AMP degradation would be expected to provide beneficial action after CNS injury.

Being the sole spatio-temporal modulators of intracellular cyclic AMP and widely distributed in brain, PDEs are also being investigated as viable therapeutic targets in neurological disorders of neuronal survival (Huntington’s disease; HD), cognition and memory (Alzheimer’s disease; AD), mood (anxiety, depression and bipolar) and thought (Schizophrenia) [28,29,30,31,32,33]. Since cyclic AMP is a critical node of many signaling pathways, including protein kinase A (PKA) and extracellular regulated kinases (ERKs) (covered below), it controls the transcriptional, translational and post-translational modifications of key molecules involved in long term potentiation, synaptic plasticity, memory consolidation and retrieval, as well as neurogenesis and neurotransmitter homeostasis [34,35]. The heritable neurodegenerative disease HD is primarily characterized by striatal neuronal death and the accumulation of misfolded mutant huntingtin protein [36]. The aggregation of misfolded protein unleashes a plethora of downstream events ranging from alterations in neuronal plasticity, axonal transport, transcriptional dysregulation and proteolytic defects [37]. Due to its key roles in synaptic plasticity, axonal transport of neurotrophic receptors, and intracellular signaling, the modulation of intracellular cyclic AMP by analogs or PDE inhibitors has become an attractive target for HD therapeutics [31]. In the neurological disorder AD, there is a dysfunction in CREB signaling, which is downstream of cyclic AMP and regulates the expression of transcription factors critical for neuronal plasticity [38,39]. The pathological sequelae of AD, neurodegeneration, synaptic dysregulation and intracellular and extracellular protein accumulation, are accompanied, in both sporadic and Mendelian AD, by the distinct post-translational processing of amyloid precursor protein and its accumulation, which triggers cyclic AMP hydrolysis that is sensitive to PDE antagonism [40,41]. Targeted inhibition of intracellular cyclic AMP hydrolysis in the AD brain can therefore provide a therapeutic approach to prevent ongoing degradation of neuronal function. In the field of psychiatric disorders of affect, mood and thought, improved understanding of genetic predisposition, stress, structural, molecular, neurochemical and synaptic alterations in key areas of the brain such as prefrontal cortex, cingulate cortex, nucleus accumbens and the medial forebrain bundle, have provided new therapeutic avenues for treatment [42,43,44]. The critical role of cyclic AMP in neuronal plasticity and intracellular signaling modulation makes its dysregulation a key event in the pathomechanisms of these disorders. Genome-wide association studies have shown that cyclic nucleotide PDEs confer susceptibility to major depressive disorders, whereas inhibitors to cyclic AMP hydrolyzing PDEs can alter outcomes in depression [45,46]. Schizophrenia, a chronic, complex neuropsychiatric disorder of cognition and thought with numerous endophenotypes [47], shows a profound disruption of neurochemical homeostasis [48]. Cognitive defects secondary to synaptic derangements and heritable molecular defects in Schizophrenia that alter axonal transport and synaptic plasticity involve changes in intracellular cyclic AMP [48]. The pharmacological therapy landscape for Schizophrenia is extensive, with more than 63 drugs under clinical investigation [49]. Included in these approaches are PDE inhibitors that are being investigated for their potential as either a monotherapy or adjunct approach to address the psychotic symptoms or cognitive defects of Schizophrenia [49,50,51].

## 2. Overview of the Cyclic Adenosine Monophosphate Signaling Pathway

### 2.1. From Membrane to Nucleus

The role of cyclic adenosine monophosphate (cyclic AMP) as a second messenger was discovered in 1956 by Earl W. Sutherland and colleagues [52]. Cyclic AMP is now known to be a pivotal component of a broad array of physiological events that include memory, immune function and metabolism [53]. These processes are controlled by extracellular signaling that potentiates cyclic AMP-mediated cellular proliferation, differentiation, plasticity, and long-term survival. Mechanisms that convert extracellular signals to gene transcription include phosphorylation cascades, proteolysis, phospholipid layer binding and calcium release, among several others. Under normal physiologic conditions, the level of cyclic AMP in a cell is regulated by a balance between the rate of its synthesis by adenylyl cyclases (ACs) and the rate of its degradation (PDEs). Cyclic AMP is generated upon stimulation of a G-type seven-transmembrane G protein-coupled receptor (GPCR) by ligand binding [54]. G protein α-subunit (G_αs_)-coupled GPCR ligands in the CNS can include neurotransmitters and prostaglandins, for example. Activation of these receptors results in the G_αs_ dissociation from the βγ subunit complex [54]. The enzyme, AC, is in turn activated by the G_αs_ subunit to catalyze the cyclization of adenosine triphosphate (ATP), thus forming cyclic AMP and pyrophosphate [55]. Currently, there are nine transmembrane AC isoforms known to be expressed in various mammalian cell types [56] and one GPCR independent soluble form of AC [34,57,58]. They largely differ in the way they are regulated by one or more of their upstream binding partners such as G protein α subunits, G protein βγ subunits, protein kinase C, calcium and bicarbonate [59,60].

Upon formation, cyclic AMP targets multiple intracellular proteins, including exchange protein directly activated by cyclic AMP (Epac), protein kinase A (PKA), small GTPases, and cyclic nucleotide-gated channels (CNGCs) [54,61,62,63,64]. The binding of cyclic AMP to protein kinase A (PKA) promotes phosphorylation of specific serine and threonine residues on various target proteins, of which the most important is a transcription factor called cyclic AMP response element–binding protein (CREB) [65]. The cyclic AMP response element (CRE) is a short palindromic sequence within the promoter region of cyclic AMP responsive genes through which gene expression is regulated. For transactivation to take place, CREB 347 is phosphorylated by PKA at serine 133 while CREB 327 is phosphorylated at serine 119 [66,67]. PKA also phosphorylates the transcription factor nuclear factor-κB (NF-κB) [68]. Whereas phosphorylation of CREB activates transcription necessary for survival of the cell [69], phosphorylation of the p50 subunit of NF-κB inhibits the transcription of pro-inflammatory cytokine genes including TNF-α and IL-1β that in turn leads to an anti-inflammatory effect [68,70,71].

### 2.2. The Role of Cyclic AMP in the CNS Axon Growth and Cell Survival

During mammalian presynaptic and postsynaptic development, neurogenesis promotes the extension of axons into target tissues, where redundant pathways undergo rearrangement and pruning through competition to enhance the efficiency of the neural network. Axons that initiate contact with target cells determine their own fate, by mediating the degree of branching and innervation. Target tissues and axons that initiate contact secrete a variety of growth factors that are essential for neuron survival, termed neurotrophic factors. When a neuron forms an association with its target tissue, neurotrophic factors exert an anti-apoptotic effect on that neuron. Although this particular process primarily occurs prenatally, few parts of the mature brain (e.g., hippocampus) do continue to grow and regenerate neurons from stem cell progenitors. The effects of neurotrophic factors on axonal growth and synaptic modulation are either facilitated by, or lead to the formation of, cyclic AMP [72,73].

Cyclic AMP has been demonstrated to mediate axon guidance, a fundamental step in the developing and regenerating nervous system. This second messenger plays a key role in regulating the polarized growth cone behaviors of neurons in response to gradients of netrin-1 or myelin-associated glycoprotein (MAG), which switch from an Epac-mediated chemoattraction during the embryonic stage to a PKA-mediated chemorepulsion during the post-natal stage [23]. This developmental switch in growth cone behavior correlates to the intracellular levels of cyclic AMP, which decrease as neurons mature [74,75]. Permanent loss of sensory and motor function following injury to the adult mammalian CNS results from a lack of regeneration. The inability of axons to regenerate is attributed to a combination of several critical factors such as decrease in the levels of neurotrophic factors [76], increased gliosis and the presence of inhibitory molecules such as chondroitin sulfate proteoglycans [77] and myelin-associated molecules [78] as well as a drop in the endogenous levels of cyclic nucleotides [24,79]. Previous work from our lab and others [24,79] have confirmed the potential for using cyclic AMP elevation as an approach to enhance axon regeneration following an injury, which appears to be directed through activation of PKA and the downstream phosphorylation of CREB. Similarly, exogenous neurotrophin supplementation, able to prime neurons for axon regeneration over inhibitory substrates such as myelin, involves the elevation of cyclic AMP via ERK-mediated inhibition of phosphodiesterases [80].

Separately, but of similar importance, are transcription-dependent processes initiated by cyclic AMP that lead to axon growth. One such signaling cascade occurs through the cytokine IL-6. Elevation of cyclic AMP can increase the production of IL-6, which is then available to form the IL-6-IL-6R receptor-GP130 functional receptor complex and activate the Janus Kinase (JAK) family of tyrosine kinases. Together with a transcription factor known as the signal transducer and activator of transcription 3 (STAT-3), the JAK/STAT signaling pathway can promote neurite growth in the presence of inhibitory myelin-associated glycoprotein (MAG) or degraded myelin [81,82]. Interestingly, blocking the IL-6 receptor or JAK failed to prevent the application of dibutyryl-cyclic AMP (db-cyclic AMP), a synthetic cyclic AMP analog, to promote neurite growth in the presence of MAG. This finding suggested that IL-6, while in part acts as an effector of cyclic AMP-mediated axon growth over myelin, it is not necessary for the db-cyclic AMP-mediated relief of myelin inhibition on axon growth [82].

Important also for axon growth are alterations in cytoskeletal assembly and polyamine synthesis, which are regulated by the Arginase-I gene [83,84,85]. Arginase-I catalyzes the conversion of arginine to ornithine, which is further acted upon by ornithine decarboxylase to form putrescine [86,87]. Overexpression of Arginase-I can overcome neurite outgrowth inhibition by myelin associated inhibitors (MAIs) such as MAG and inhibiting Arginase-I in DRGs blocks the effect of cyclic AMP on axon regeneration [85]. That Arginase-I was expressed in cerebellar neurons in response to exogenous db-cyclic AMP or brain derived neurotrophic factor (BDNF) was also demonstrated in other studies [83,85]. Recently, early or late delivery after SCI of putrescine, a catabolic product in Arginase-I pathway, was shown to supplement the positive outcomes of Schwann cell transplantation and improve sensory and serotonergic axon regeneration [88]. Moreover, spermidine, a downstream catabolic product of putrescine, was also shown to be effective in promoting DRG neurite outgrowth and overcoming axon growth inhibition by MAIs [89]. These findings suggest that the Arginase-I mediated polyamine pathway could play a critical role in traumatic SCI and TBI and therefore be an attractive therapeutic target for axon regeneration. Many pathways that regulate the transcription of Arginase-I also regulate intracellular cyclic AMP levels [90,91,92]. In fact, cyclic AMP has been reported to play a permissive role in the transcription of Arginase-I in a C/EBPβ dependent manner [93]. Cyclic AMP has been shown to work synergistically with other intracellular signaling pathways, e.g., STAT6, to promote Arginase-I transcription. This permissive role of cyclic AMP can be clearly exploited for therapeutics aimed at promoting regeneration and repair of the damaged CNS. Interestingly, a high-throughput screen for Arginase-I transcription regulators identified a molecule, Daidzein, as a non-canonical enhancer of Arginase-I expression [94]. Subcutaneous Daidzein, when delivered at the time of stroke in a rat model, was shown to improve behavioral outcomes [95].

Until the discovery of Epac, the downstream effects of cyclic AMP signaling were only attributed to protein kinase A (PKA) activation [61,96,97,98,99,100,101]. Canonically, cyclic AMP’s binding to Epac targets it to the plasma membrane and activates Ras family members, Rap1 and Rap2 [102]. Therefore, spatio-temporal activation of Epac is tightly regulated by the cyclic AMP-PDE network. Epac also has additional effectors such as c-Jun-N-terminal kinase (JAK) and phospholipase D [103,104]. Cyclic AMP-dependent neuronal excitability and resting membrane potential in cerebellar granular neurons has been shown to be mediated by Epac-Rap1-p38MAPK, in a PKA-independent manner [105]. In fact, Epac can oppose PKA-mediated inhibition of Akt by stabilizing Akt association with AKAP150 and promoting Akt activation [106]. Thus cyclic AMP can activate the cell survival mTOR pathway via Epac and also regulate output of the mTOR pathway via Rheb/PDE4D [107]. Discerning biological roles of Epac has been possible due to the development of PKA and Epac specific inhibitors. Epac directly regulates neuronal polarization via Rap1B [108]. Epac has been shown to enhance neurite outgrowth and to switch the proliferation phase of PC12 cells to differentiation [109,110]. Apart from its effects on neurite growth, Epac has been shown to be a presynaptic effector for cyclic AMP mediated synaptic potentiation in excitatory neurons and synapses [111,112]. The critical role of cyclic AMP-Epac is exemplified by an absolute necessity of Epac in the hippocampus for the contextual memory retrieval task [113]. Interestingly, the Epac-p38-MAPK pathway has also been recently implicated in pituitary adenylate cyclase-activating polypeptide (PACAP)-mediated long-term depression in CA1 hippocampal neurons [114]. Aside from synaptic modulation, neurotransmitter release and its effects on memory and learning, Epac’s role in the fields of vascular biology, inflammation, blood–brain barrier integrity and myelination have also been investigated [23,64,112,113,115,116,117]. Further understanding of the neuroprotective and neuroregenerative role of cyclic AMP-Epac in neurological disorders and neurotrauma is expected when the new generation of Epac selective agonists and antagonists can be employed in experimental paradigms [118].

Conversely, when intracellular levels of cyclic nucleotides are lowered, anti-apoptotic signaling mechanisms can be hindered to produce cell death via pro-apoptotic factors [119]. Increases in cyclic AMP have been shown to rapidly recruit tyrosine-kinase B (TrkB) receptors to the surface of cell membranes. TrkB, when bound by BDNF [120], leads to the activation of the phosphatidylinositol-3-kinase (PI3K)/Akt cascade to stimulate the simultaneous expression of “bcl-2” and inactivation of “bcl-2 associated death promoter” (Bad), anti-apoptotic and pro-apoptotic proteins, respectively [121]. Thus, cyclic AMP, along with the Ras-MAPK pathway, plays an important role in the switch between Bad and bcl-2 to control pro-apoptotic and anti-apoptotic signaling in the cell [122,123,124,125].

## 3. Overview of Cyclic Nucleotide Phosphodiesterases

### 3.1. The Phosphodiesterase Family

Soon after the discovery of cyclic AMP, Butcher and Sutherland were the first to identify the enzymes responsible for cyclic AMP degradation, termed phosphodiesterases [126]. The breakdown of cyclic AMP and cyclic GMP to 5′ AMP and 5′ GMP, respectively, is catalyzed by PDEs via the hydrolysis of their 3′ phosphate bond [127]. In doing so PDEs reduce the intracellular concentrations of cyclic AMP and cyclic GMP and thus act as a terminal switch to their respective downstream signaling cascades. Recent work by Krishnamurthy et al. [128] has demonstrated that PDEs play an essential role in the termination of the cyclic AMP signaling cascade by catalyzing its hydrolysis and dissociation, which is otherwise bound with high affinity to the regulatory subunit of PKA. This dissociation thus resets cyclic AMP-mediated signaling to its basal level. Moorthy et al. [129] have proposed a mechanism for PDE-mediated hydrolysis of cyclic AMP when bound to its downstream effector, PKA: with cyclic AMP binding to the PKA-regulatory subunit, the catalytic subunit of PKA dissociates from the holoenzyme to mediate downstream signaling effects while binding of PDE to the catalytic region of PKA releases cyclic AMP. The cyclic AMP molecule is then immediately hydrolyzed to 5′ AMP. The PKA R- and C-subunits reassemble via another binding site, precipitating the inactive form of the PKA holoenzyme and promoting the subsequent interruption of downstream cyclic AMP signaling [129].

Cyclic nucleotide PDEs make up a large family of enzymes that degrade cyclic nucleotides (i.e., cyclic AMP and cyclic GMP) and thus regulate their intracellular concentrations. Mammalian PDE enzymes are divided into 11 families of proteins, encoded by 21 genes that are transcribed to more than 100 messenger RNA transcript-variants, most of which are translated into proteins. The multiplicity and complexity of these enzymes are attributed to their extensive mRNA alternative splicing, diverse *cis*-/*trans*-regulatory elements and translational regulation, which collectively produce species with different catalytic properties, intracellular localization, tissue specificity, signaling pathway modulation and sensitivity to PDE inhibitors [130,131,132,133]. Phosphodiesterase enzymes share structural features that are common among all isoforms and that are highly conserved across species. These consist of a regulatory domain in the N-terminus, a conserved catalytic region (which catalyzes cyclic nucleotides) downstream of the regulatory domain, and a region downstream from the catalytic region and upstream of the C-terminus which, in some instances, is prone to prenyl modification, as in PDE6, or phosphorylation by MAPK, as in PDE4 [134,135,136,137]. PDEs though can be divided into three broad groups based on their catalytic selectivity to cyclic nucleotides: cyclic AMP-specific PDEs (PDE4, PDE7, and PDE8), cyclic GMP-specific PDEs (PDE5, PDE6, and PDE9), and PDEs that lack specificity, hydrolyzing both cyclic nucleotides (PDE1, PDE2, PDE3, PDE10, and PDE11), though they do exhibit a higher affinity to one cyclic nucleotide. This specificity has been proposed to be associated with a “glutamine switch” mechanism in the catalytic region that is responsible for the recognition and selectivity for cyclic nucleotides. A highly conserved glutamine residue within the binding pocket that regulates the binding of the cyclic nucleotide purine ring has been proposed to be the key to the specificity of PDEs towards their substrates [138,139]. In selective PDEs, glutamine’s rotation is restricted by the adjacent residues which restricts binding either to cyclic AMP or cyclic GMP based on its position. However, that the glutamine residue is the sole determinant of PDE substrate specificity remains controversial. Among the PDE family of enzymes, those belonging to the PDE4 subfamily are the best characterized, due to their role as the main negative regulator of cellular cyclic AMP in the CNS [35], their key activity in maintaining cyclic AMP signal compartmentalization [140,141] and their implicated involvement in a wide range of neurological diseases and injury [30,45,142,143,144,145,146,147].

### 3.2. The Structure and CNS Distribution of Phosphodiesterases

All the 21 widely known members of the phosphodiesterase family have a conserved catalytic domain at the C-terminal. Dual cyclic nucleotide PDEs have either a Ca^2+^/CAM binding domain (PDE1A, 1B, and 1C) or GAF-A/B domain (PDE2A, 10A, and 11A) or a transmembrane domain (PDE3A, 3B) as their N-terminal regulatory domain. Cyclic GMP-specific PDEs have either no specific regulatory domain (PDE9A) or exhibit a GAF-A/B domain (PDE5A, 6A, 6B, and 6C) at their N-terminal. The PDE6 family (6A, 6B, and 6C) has additional components such as prenyl groups, a δ-subunit and a γ-subunit. In contrast, cyclic AMP-specific PDEs have either a conserved stretch of amino acids (UCR1, UCR2) at their N-terminal region (PDE4A, 4B, 4C, and 4D) or possess a PAS domain (PDE8A, 8B) or have no specific regulatory domains (PDE7A, 7B). However, multiple PDE variants that utilize a different coding exon or undergo splicing often end up shorter than the long forms mentioned above. These short or super-short PDE isoforms have minimized regulatory domains and very rarely, a truncated catalytic domain (PDE4B4).

PDEs undergo post-translational modification by way of enzymatic and covalent processes that generally include, among other things, phosphorylation, prenylation, and ribosylation. These modifications are expected to serve as feedback signals to spatio-temporally regulate the activity of PDEs. In fact, phosphorylation is one of the important methods of activation of PDE regulatory domains. For example, in PDE1A, PKA-mediated phosphorylation of Ser120 determines CaM binding sensitivity, whereas for PDE1B, CaMK-II mediated phosphorylation can dramatically decrease the CaM binding affinity up to six fold [148,149]. In the PDE3 family, the membrane insertion domain (NHR1) and the membrane targeting (NHR2) domains are separated by phosphorylation sites (S273, S296) that are believed to be important for protein-protein interactions and activity regulation. These inter-domain phosphorylation sites in PDE3s are responsive to IGF-1, insulin and cyclic AMP modulators [150]. The regulatory domain in PDE5 has PKA/PKG phosphorylation sites and auto-inhibitory elements, whereas there is the presence of a GAF-A/B ligand binding domain that is highly specific to cyclic GMP over cyclic AMP [151]. In contrast, in dual cyclic nucleotide-specific PDEs (PDE10, 11), cyclic AMP binding to the GAF-A/B domain might lead to altered PDE activity by allosteric mechanisms [152]. The γ-subunit of the PDE6 family is inhibitory in nature and has multiple sites for posttranslational modifications (phosphorylation, ADP ribosylation) that could modulate the interaction of the γ-subunit with a heterotrimeric G-protein called transducin, which relieves the inhibition on the catalytic domain of PDE6 [153,154,155,156]. PDE4 isoforms have unique upstream conserved regulatory regions (UCR1 and UCR2). PDE4 enzymes can be subcategorized into three groups based on the presence or lack of certain UCRs; long isoforms have UCR1 and UCR2 whereas short isoforms are missing UCR1 and super-short isoforms contain a truncated UCR2. UCRs regulate the activity of PDE4s by modulating their phosphorylation by PKA and ERK [157] as well as dimerization [158]. Cyclic AMP activation of protein kinase A (PKA) leads to the phosphorylation of UCR1 at a consensus site (RRESF) in a PKA-dependent manner, which alters the UCR1–UCR2 interaction, and activates PDE4 [133,159,160,161]. With the exception of PDE4A, ERK phosphorylates the C-terminus of all PDE4 isoforms, resulting in the induction or inhibition of short and long PDE4 isoforms, respectively. That is to say, PDE4 regulation by ERK and PKA is modulated by the presence or absence of UCR1 [162]. In addition to specifically hydrolyzing cyclic AMP, the N-terminus region of PDE7A1 has repeat sequences that bind to PKA and inhibit its kinase activity. PDE7A1 thus acts as a bi-functional inhibitor of cyclic AMP signaling [163]. In contrast, with no intact repeat sequences, PDE7A2 is not expected to function as a PKA inhibitor. Both PDE7B1 and PDE7B3 carry a predicted PKA phosphorylation site on their N-terminus, however the functional consequences of phosphorylation at that site are yet to be understood [164]. Finally, cyclic AMP-specific PDE8 contains N-terminal REC and PAS regulatory domains that can modulate PDE8 activity, either by assisting in conformation stability, protein-protein interaction or its association with cofactors. In addition, PDE8 also has myristolation and multiple phosphorylation sites in the N-terminal region, a REC domain and a region between the PAS and catalytic domains [165,166].

Almost all PDE isoforms are expressed in the CNS with an exception of PDE4C. The PDE6 family is specifically expressed in retinal system. In the cerebellum, there is predominant expression of cyclic AMP responsive PDEs (PDE4, 7 and 8), whereas, in the olfactory bulb, a mixture of dual specificity PDEs (PDE1, 2, and 3) and the cyclic AMP specific PDE4 family are present. Striatum and amygdala conspicuously do not express PDE3B [167].

## 4. Pathological Alterations to Phosphodiesterases and Cyclic Nucleotides

### 4.1. Phosphodiesterase and Cyclic Nucleotide Changes Following Neurotrauma

CNS injury initiates a cytotoxic pro-inflammatory cascade and leads to neuronal excitotoxicity through the extracellular release of the neurotransmitter, glutamate [168,169,170]. Neurons, astrocytes, and activated microglia can also produce pro-inflammatory cytokines [171,172], though current understanding suggests that activated microglia are the predominant source of TNF-α and IL-1β [173,174]. In fact, these two cytokines are known to reduce intracellular cyclic AMP specifically within microglia and they are thought to do so primarily by way of an upregulation of cAMP-specific PDE expression and activity [175]. Following experimental TBI, studies have shown that microglia rapidly release large quantities of TNF-α and IL-1β [176,177,178,179,180]. This release occurs concurrently with a simultaneous rise in glutamate and a reduction in intracellular cyclic AMP that is accompanied by alterations in glial cell function, [181,182,183,184,185]. These and other cytotixic mediators initiate an influx of calcium into neurons by way of NMDA receptor activation. The NOS signaling pathway is activated and ultimately mitochondrial dysfunction leads to apoptosis [170,186]. Ghosh et al. recently characterized the signaling pathways responsible for reductions in the concentrations of intracellular cyclic AMP in microglia following exposure to cytokines such as TNF-α. Upon cytokine challenge, microglia showed an increased immunoreactivity for iNOS and COX-2, enhanced phosphorylation of ERK1/2 and NF-κB-p65 as well as the degradation of IκB and NF-κB-p65 nuclear translocation. These signaling changes in response to cytokines were perturbed when intracellular cyclic AMP concentrations remained high [187]. Microglia, when exposed to disparate extracellular effectors may undergo immunophenotypical changes that alter their functional roles in the injured CNS from their normal surveillant behavior, whether deleterious or reparative, [188]. Microglia provide a supportive role for repair when stimulated with anti-inflammatory cytokines, such as interleukins (IL)-4, 10 and 13 as well as tumor growth factor-β (TGF-β) [189]. In this state microglia release angiogenic and regenerative growth factors including vascular endothelial growth factor (VEGF), platelet-derived growth factor (PDGF), and brain-derived neurotrophic factor (BDNF), which creates a supportive environment for oligodendrocyte maturation, remyelination and axon growth [190]. Ghosh et al. recently demonstrated that immunophenotypic conversion of microglia from a deleterious to pro-reparative form, as recognized using the prototypical markers iNOS and Arg1, respectively, required the combined presence of anti-inflammatory cytokines IL-4 or IL-13, and elevated intracellular cyclic AMP, both in culture and in vivo following acute SCI [191].

At the cellular level, injury to mature non-proliferating neurons promotes the aberrant stimulation of a variety of signaling cascades, often invariably triggering the process of programmed cell death. Neurodegeneration and cellular injury activates the downstream expression of cyclin D1, in part through an activation of PDE. Chen and colleagues [192] demonstrated that the neurodegenerative effects associated with PDEs are linked to glutamate-stimulated cyclin D1 expression and the promotion of caspase-3 activity. In combination with the pro-apoptotic activity of caspases, PDE activation is associated with the release of cytochrome C and the inhibition of PI3K and Akt [193]. Ray et al. demonstrated that caspase-3 inhibition is essential for preventing glutamate-induced apoptosis in the brain [194]. Ghosh et al. [175] showed that inflammatory cytokines released by activated glial cells following injury act through PDEs to disrupt cellular homeostasis maintained by intracellular cyclic AMP. The inhibition of PDEs therefore not only serves to disrupt the neurotoxic feedback loop of secondary injury and cell death but is also important for the initiation of neurogenesis and neurorepair.

Unlike the peripheral nervous system (PNS), CNS regeneration is further inhibited by Nogo-A, myelin-associated glycoprotein (MAG), and oligodendrocyte-myelin glycoprotein (OMgp), collectively known as MAIs [78,195,196,197,198,199,200]. MAG, Nogo and OMgp are not homologous in structure or sequence yet all can bind a single receptor, the Nogo receptor (NgR1). Upon binding to NgR1, the MAIs impede axonal growth by promoting growth cone collapse [201,202]. It is known that MAG, Nogo and OMgp, produced mainly by oligodendrocytes, stimulate Rho-A, a member of the small GTPase family. Rho-A in turn activates Rho-Kinase (ROCK) and thus inhibits axonal cytoskeletal assembly [203]. Chondroitin sulfate proteoglycans (CSPGs), secreted by reactive astrocytes, also result in Rho-A activation via the membrane protein tyrosine phosphatase PTPσ [204]. Rho activation is inhibited by cyclic AMP-PKA and subsequently PKA-mediated phosphorylation of activated-Rho inhibits its interaction with ROCK [204,205,206,207].

Thus, when the CNS is damaged, activation of the inflammatory cascade forms a positive feedback loop of neuronal injury that serves to exacerbate the initial damage and dysfunction. One target of this cascade is the reduction of intracellular cyclic AMP through PDE-mediated hydrolysis, disrupting cellular homeostasis and suppressing a range of signaling molecules involved in neuronal survival, neural plasticity, neurogenesis, regeneration and remyelination [21,22,27,208]. As understanding of these pathways continues to expand, promising therapeutic strategies focused on antagonizing PDE activity and restoring intracellular cyclic AMP to promote neuroprotection and neurorepair have shown benefit in experimental models of neurotrauma.

### 4.2. Phosphodiesterase and Cyclic Nucleotide Alterations in Neurodegenerative Conditions

Neurodegenerative disorders share features of neuronal cell death and the inevitable loss of CNS architecture, which results in clinical manifestations of neural dysfunction. Some of the most extensively characterized neurodegenerative conditions include Alzheimer’s disease (AD) and Amyotrophic Lateral Sclerosis (ALS), movement disorders such as Huntington’s disorder (HD) and Parkinson’s disease (PD) as well as psychiatric illnesses such as Major Depressive Disorder and Schizophrenia.

Alzheimer’s disease is likely the most studied of the aforementioned neurodegenerative illnesses. It is generally defined as a progressive, age-related loss of cognitive function and memory. Although classically described as brain tissue atrophy accompanied by the presence of β-amyloid plaques and neurofibrillary tangles, AD is now also known to be associated with the increased CNS expression of numerous PDE mRNA species, including PDE2, PDE4A, PDE4B, PDE5, PDE7 and PDE8B as demonstrated in AD human brain tissue and rodent experimental models [28,209,210,211,212,213]. Early stages of AD are associated with brain tissue expression of PDE4A, PDE4B, and PDE7A, while at more clinically severe stages, increases in PDE8B are observed [214]. With preclinical research suggesting that PDE4D is particularly important in effects on cognition, this particular isoenzyme has been studied in the hippocampus of a patient with AD where the isoforms PDE4D1 and PDE4D2 were found to be increased [215]. PDEs have also been implicated in the pathologic mechanisms described clinically in movement disorders, namely Huntington’s disease (HD) and Parkinson’s disease (PD). HD is characterized by cognitive deficits accompanied by chorea, motor symptoms classically described as involuntary writhing movements [216,217,218]. PD, on the other hand, is defined by slowness, rigidity, and a classically described pill-rolling tremor. Both illnesses however demonstrate striatal neurodegeneration. In the striatum, one of the most studied and abundantly expressed of the PDEs is PDE10A [219]. PDE10A was found localized to the membranes of dendrites as well as the dendritic spines of striatal GABAergic interneurons [220]. For example, Hebb et al. revealed significantly lower levels of PDE10A in the subcortical structures of post-mortem HD patients (i.e., caudate nucleus, putamen, and nucleus accumbens) in comparison to controls [221]. Moreover, early cognitive deficits in HD could also be causally related to hyperactivity of cyclic AMP signaling. This notion is supported by hippocampal PKA hyperactivity in people with HD, in which there is inactivation or reduced activation of PDE4 isoforms [222]. On the other hand, PDE1B could also be potentially involved in striatal neurodegeneration. Expressed in a similar pattern as PDE10A in the brain, PDE1B is instead localized to spiny interneurons, suggesting that PDE1B is specific to neurons involved in dopaminergic signaling [219,223]. The differences between opposing signaling cascades in the striatum is highlighted by the opposing effects of PDE1B and PDE10A on motor behavior. PDE10A and PDE1B are predominantly expressed in the striatopallidal D2 indirect pathway, and the striatonigral D1 direct pathway, respectively. Stimulation of D2 receptors leads to decreased levels of cyclic AMP, secondary to negative coupling to AC, while stimulation of D1 receptors leads to increased levels of cyclic AMP, secondary to AC activation. In this way, stimulation of D2 leads to behavioral inhibition while that of D1 leads to behavioral activation [224].

Although neuronal death is the common denominator among the aforementioned neurodegenerative disorders, psychiatric illness is not commonly associated with atrophied brain tissue. Instead, our understanding of psychiatric illness has most commonly described dysfunction of neural networks and/or neurotransmitter imbalance. However, Major Depressive Disorder (MDD) is indeed a psychiatric illness that can be described as neurodegenerative in nature because of the commonly cited presence of hippocampal dentate gyrus and CA3 atrophy [225,226,227,228,229]. Like in HD, in MDD there is evidence of a significant reduction in the signaling activity of proteins associated with cyclic AMP, such as AC, PKA, and CREB [230,231,232,233]. Specifically, enhanced PDE4 and PDE11 have been reported to be associated with MDD [234]. Through studying genotyped single-nucleotide polymorphisms (SNPs) in the PDE superfamily of patients with MDD, Wong et al. among others, found strong association loci within PDE9A and PDE11A for the diagnosis of MDD [46]. Moreover, CNS levels of the PDE11A isozyme have been shown to respond to antidepressant treatment [235]. Interestingly, O’Donnell and Xu have suggested that decreased norepinephrine and 5-HT neurotransmission in MDD patients may result in attenuated cyclic AMP signaling that could lead to a compensatory reduction in PDE4 levels [33].

## 5. The Utility of PDE Inhibitors for Neuroprotection and Neurorepair

PDE inhibition as a therapy to repair the damaged or diseased CNS has been studied in various pathological conditions. One of the most promising strategies to achieve this is by administering selective pharmacological antagonists [33,236,237,238].

### 5.1. PDE1

Gomez et al. demonstrated that the PDE1 inhibitor Vinpocetine decreased cerebral inflammation by reducing the expression of TNF-α and IL-1β [239]. In work by Molnar and Gaal, Vinpocetine could also enhance long-term potentiation in the rat dentate gyrus [240]. Vinpocetine was also shown to enhance memory [241], modulate oxidative stress and reverse intracerebroventricular streptozotocin-induced cognitive dysfunction in rodents [242], and in humans, enhance cognitive behavioral test results [243]. A study by Zaitone et al. demonstrated the effectiveness of Vinpocetine as a neuroprotective agent in rotenone-induced Parkinsonism. Treated rats showed an improvement in locomotor function and exhibited substantially decreased levels of malondialdehyde (MDA) and glutathione (GSH) [244]. Moreover, Vinpocetine was shown to induce a noticeable functional improvement in a 3-Nitropropionic acid-treated HD rat model [245], and to completely restore hippocampal cyclic AMP levels and normal motor function, reversing locomotor hyperactivity, in a fetal alcohol spectrum disorder (FASD) mouse model, suggesting the potential use of Vinpocetine in FASD treatment [246]. Laddha and Bhatnagar investigated novel compounds with a dual anti-inflammatory and PDE1 inhibition effects and suggested their potential importance in PD. However, the authors implied that more studies were required to confirm a role of PDE1 inhibition with these agents as a therapy for PD [247]. Furthermore, Amantadine, an agent used in PD also exhibits PDE1A inhibition, which further suggests the possibility of a role for PDE1 in PD [248].

### 5.2. PDE2

PDE2 inhibition has also been shown to have effects on cognitive function. Domek-Lopacinska and Strosznajder demonstrated the effects of the PDE2 inhibitor BAY 60-7550 on learning and memory processes in the aged mouse by ways of enhancing neuronal nitric oxide synthase (nNOS) activity in the brain [249]. It is also noteworthy that NOS has been shown to play an important role in brain damage resistance and a reduction of secondary injury after brain trauma [250]. Studies by Terpolilli et al. showed the significant role of NOS and the NO-cGMP pathway in decreasing and preventing brain damage in ischemic stroke, subarachnoid hemorrhage, as well as traumatic brain injury [250,251,252,253]. PDE2 inhibition by BAY 60-7550 has also been shown to improve tryptophan-depletion-induced memory deficiency [254] and reverse scopolamine or MK-801-induced [254] memory deficits in rats as well as improve memory in an AD mouse model [255].

### 5.3. PDE3

PDE3 inhibition showed neuroreparative effects following trimethylin (TMT)-induced neuronal loss in the mouse hippocampal dentate gyrus. Treatment with the PDE3 inhibitor Cilostazol led to elevations in CREB and an improvement in depression-like symptoms in mice undergoing the forced-swimming test [256]. The same study found that chronic (2 weeks) but not acute (2 days) treatment of mice with Cilostazol led to the proliferation of endogenous neural progenitors and an increased number of immature neurons, specifically in the granule cell layer of the hippocampus. Yanai et al. demonstrated that Cilostazol enhanced context-dependent long-term fear memory in mice by utilizing the, “Morris water maze probe test” (MWM). In their study, Cilostazol notably improved the number of phosphorylated-CREB-positive cells in the mouse hippocampal dentate gyrus, reflecting the effects of this PDE3 inhibitor on memory and learning through increasing intracellular cyclic AMP in the hippocampus [257]. A study by Hiramatsu et al. revealed the effects of Cilostazol on amyloid β peptide (25–35)-induced memory impairment in mice. Cilostazol prevented an increase in malondialdehyde (MDA) levels suggesting a mechanism of action associated with reduced oxidative stress in the frontal cortex and hippocampus [258]. Currently, there are ongoing human clinical trials employing Cilostazole in ischemic stroke (Phase IV), AD (Phase II) and Dementia (Phase II) based on the efficacy achieved in corresponding animal models (Table 1).

### 5.4. PDE4

The first generation PDE4 inhibitor Rolipram was shown to have neuroprotective and neuroregenerative effects following SCI [22,24,25,37,259,260,261]. When used in combination with the cyclic AMP analog, db-cyclic AMP, and Schwann cell grafts, Rolipram was shown to improve anatomical and functional outcomes [24], including significant tissue sparing, increased axonal growth and myelination by the transplanted Schwann cells, and improved functional locomotor recovery [24]. Subcutaneous or intravenous Rolipram delivery has been shown to improve axonal growth within an inhibitory milieu of degraded myelin proteins [25]. Similar effects were observed in vivo when Rolipram was administered subcutaneously as a combination therapy with embryonic spinal cord tissue transplanted into injury site in a spinal cord hemisection model. Immunohistochemical staining for serotonergic axons and reactive astrocytes showed very few 5-HT positive axons within the transplant of vehicle-treated and untreated animals and reactive astrocytes surrounding the transplant. In Rolipram-treated animals there were many more serotonergic axons that had grown into the transplant [25]. The forelimb paw placement test showed that the Rolipram group had a higher functional score, which was attributed to the regenerating axons. The Rolipram-treated group also showed reduced glial fibrillary acidic protein (GFAP) expression compared to controls, indicating decreased glial scar formation [25]. In a recent study by Costa and colleagues [260], the effects of continuous Rolipram administration for two weeks, given alone in a moderate T10 contusion rat model, were assessed. It was revealed that Rolipram treatment improved locomotor recover. A significant reduction of external paw rotation was demonstrated during treadmill walking in Rolipram-treated animals after eight weeks of therapy. Rolipram administration also resulted in greater white matter sparing [260]. Moreover, a study by Whitaker et al. revealed the protective effects of Rolipram on spinal cord oligodendrocytes following cervical spinal cord contusion. PDE4A, -B, and -D expression in oligodendrocytes was confirmed, and cell quantification showed decreased oligodendrocyte apoptosis beginning at 24 h post-injury through endpoint at 72 h with Rolipram [261]. Specifically, in the acute SCI setting, Rolipram has been shown to antagonize the production of PDE4B1, PDE4A5 and the phosphorylation of PDE4A5, as well as reduce the expression of MCP-1 and the spinal tissue infiltration of immune cells [259]. Bao and colleagues demonstrated the anti-inflammatory and anti-oxidative effects of a selective PDE4 inhibitor, IC486051, when administered in the first three days following a moderately-severe thoracic-clip-compression SCI. Treatment with IC486051 reduced expression of gp91 (phox), nitric oxide synthase, and cyclooxygenase-2 as well as antagonized the formation of free radicals when compared to controls. The treatment group also showed significant locomotor recovery 1–2 months following SCI as measured using the Basso, Beattie, and Bresnahan (BBB) score, and decreased hind paw mechanical allodynia. The effects also included white matter sparing at two months post-SCI [262]. Schaal et al. sought to identify the optimal dose, route and therapeutic window for Rolipram delivery after acute contusive SCI [259]. It was found that a dose of 1.0 mg/kg when delivered intravenously was the most effective, whereas the therapeutic window extended through 48 h following SCI, with maximal neuroprotection noted at 2 h after injury [259]. A significant improvement in locomotor function was noted when employing the optimal administration parameters [259]. Furthermore, Rolipram has been shown to improve the effectiveness of cell therapies when employed in models of SCI [24,263].

A study by Jindal et al. revealed the antidepressant-like effect of the PDE4 inhibitor Etazolate following olfactory bulbectomy (OBX). After OBX the experimental rats showed an elevation in corticosterone levels, a reduction in BDNF and phosphorylated CREB levels, and manifested depression-like symptoms. Etazolate treatment attenuated behavioral symptoms and reduced the OBX-induced changes in BDNF, CREB, and cyclic AMP levels in comparison to the OBX non-treated group [264]. It was shown by Bruno et al. that the PDE4D inhibitor (GEBR-7b) enhanced object and spatial memory performance in the object recognition tests. When administered to rats and mice in doses that did not induce non-desirable emetic side effects, GEBR-7b was shown to be 3–10 times more potent than Rolipram, which induced emetic signs at minimal potent doses [265]. Following chronic treatment with GEBR-7b in an AD mouse model, there was a significant improvement in spatial memory [266]. Rutter and colleagues demonstrated the potency of the PDE4B inhibitor GSK356278 towards reducing anxiety while improving cognitive function in various mammalian species with no side effects under the experimental conditions examined [267]. Using a radial maze for analysis of the inhibitory avoidance task in rats, Zhang et al. showed that PDE4D inhibition promoted improvements in both working (WM) and reference memory (RM) that were originally negatively affected by MK-801-induced NMDA receptor antagonism. MK-801-induced amnesia, as evaluated using the passive avoidance behavior test, was also significantly reduced with PDE4D inhibition [33]. Barad and colleagues showed that Rolipram increased long-term retention in freezing to context in mice, a function regulated by the hippocampus as well as improved memory [236]. Gallant et al. discovered a potent selective PDE4 inhibitor, MK-0952, which was effective in enhancing long-term memory and cognitive improvement [268]. Moreover, at non-emetic doses, L-454560, a potent and competitive inhibitor of PDE4 isoforms (A, B and C) that was originally designed for the treatment of asthma, was shown to enhance memory function as measured using the “delayed matching to position (DMTP)” version of the MWM [269]. A PDE inhibition study, assessing effects on cognition in adult male cynomolgus monkeys using an object retrieval test, showed that both Rolipram and Sildenafil improved function. Treated monkeys had performance improved from 50% to 70% correct reaches [270]. A study by Imanishi et al. evaluated the therapeutic effects of Rolipram on induced learning and memory deficits utilizing a passive avoidance task in mice and a three-panel runway paradigm in rats. Cognition was affected experimentally by three methods: four-vessel-induced ischemia, electric convulsive shock, and the administration of Scopolamine. Results showed that Rolipram improved the three-panel runway performance and reversed the avoidance response impairment in rodents in a dose-dependent manner [271].

Administering PDE4 inhibitors to C57BL/6 mice has been shown to reduce MPTP-induced dopamine reduction in the striatum. PDE4 inhibition also showed a neuroprotective effect that was reflected by an increased survival of hydroxylase-immunopositive neurons in the substantia nigra [272]. The neuroprotective role of PDE4 inhibition on dopaminergic neurons was also demonstrated by Yamashita and colleagues where Rolipram significantly improved the survival of neurons following a Forskolin challenge in vitro [203] and by Yang et al. [273]. Collectively these results provide support for PDE4 inhibition as a promising therapeutic strategy in PD. In a work by DeMarch and colleagues, it was reported that PDE4 inhibition can provide benefit in an experimental paradigm of HD. Rolipram-treated R6/2 mice (with a daily dose of 1.5 mg/kg initiated at the fifth postnatal week) showed longer survival and manifested less neurological symptoms when compared to the vehicle-treated control group. Histological and immunohistochemical analysis showed decreased microglial activation, improved neuronal morphology and size in the striatum, increased volume and decreased atrophy of the brain and striatum, respectively. These findings were complemented by a prominent increase in CREB activation and BDNF levels in striatal spiny neurons [274]. These results built upon an earlier study where Rolipram’s therapeutic effects in HD were studied utilizing a quinolinic acid induced-striatal excitotoxicity rat model [275]. Moreover, Rolipram demonstrated effectiveness in sparing parvalbuminergic interneurons and restoring functional activity and motor coordination in R6/2 mice by blocking the sequestration of CREB into striatal neuronal intranuclear inclusions [276]. These findings provide evidence of the therapeutic benefit of PDE4 inhibition for HD. Rolipram has been reported to also reduce haloperidol-induced dyskinetic movements in rats as haloperidol binding to D2 receptors reduces cyclic AMP. In these investigations Rolipram elevated cyclic AMP levels, increased dopamine D2 receptor density in the striatum, and improved behavioral function, acting in a dose-dependent manner, providing a potential use for Rolipram as a therapy in Tardive Dyskinesia (TD) [277].

Approval for use of the next generation PDE4 inhibitor HT-0712 is currently being sought for conditions of memory loss, as it has been shown to enhance motor recovery and cognition through cortical restructuring after ischemia [278]. A Phase IIa clinical study was carried out in elderly subjects with an age-associated memory impairment by Dart Neuroscience [279]. Enrolled patients were provided HT-0712 for 28 days, which was reported to be well-tolerated, with no effects on short term memory or passive EEG. However, the clinical study observed that a single dose of HT-0712 led to a significant change in long-term memory of word list recall. PDE4 inhibitors may also soon be translated for SCI and TBI clinical trials based upon the therapeutic profile of PDE4 inhibitors experimentally [259,280].

### 5.5. PDE5

It has been reported that administering the PDE5 inhibitor Sildenafil can reverse cognitive impairments in mouse models [29,281,282]. In evaluating the efficacy of Sildenafil in reversing the cholinergic-muscarinic antagonism-induced memory and learning deficit, Devan and colleagues showed that Sildenafil citrate inhibited scopolamine-induced cognitive impairments, an effect that was demonstrated utilizing the 14-unit T-maze test in rats [283]. In another study by Devan et al., aimed at evaluating the effects of PDE5 inhibition on enhancing age-related memory deficits, it was shown that a significant improvement in long-term information retention could be obtained. In this investigation, rodents were subjected to T-maze as well as Morris water maze testing. Neither Sildenafil nor Vardenafil, given in the T-maze and water maze experiments, respectively, had any positive cognitive performance effects in the first acquisition test. However, after an injection-free period of seven days, the PDE5-inhibition group showed a significant improvement in memory retention, compared to control groups. These results demonstrated the potential of PDE5 inhibitors in antagonizing age-associated memory deficits, such as dementia [284]. It is also important to note the significant effects of PDE5 inhibition on facilitating neuroprotection through the enhancement of the NO-cGMP pathway. Charriaut-Marlangue et al. studied the neuroprotective effects of Sildenafil administration in an induced hypoxic ischemia neonatal rat model. Selective PDE5 inhibition resulted in an enhancement of cerebral blood flow, mitigating ischemic damage as well as improving open-field locomotor function. Interestingly, Sildenafil also reduced apoptosis, astrocytosis, and microgliosis [285]. Additionally, it has been shown that inhaling NO antagonizes hyperoxia-induced white matter damage, mitigates induced-stroke-associated brain damage, enhances CNS myelination, and promotes neural progenitor proliferation in various neonatal models [285,286,287,288,289].

Another neurodegenerative condition in which PDE5 inhibition was shown to be effective is HD. A 3 mg/kg dose of Sildenafil administered intraperitoneally to R6/1 mice immediately following training led to the elevation of cyclic GMP levels with a concomitant cognitive enhancement on the novel object recognition and passive avoidance tests [290]. PDE5 inhibitors Sildenafil and Vardenafil were shown to exhibit neuroprotective properties in a HD rat model following 3-nitropropionic acid-induced neurotoxicity, by modulating BDNF, CREB, and Calpain leading to striatal neuronal survival. The effects were demonstrated by increased DARPP-32 levels in the striatum, decreased Calpain activation and lesion volumes, and improved behavioral function in treated rats [291].

Garcia-Barroso et al. reported on the efficacy of Tadalafil, another PDE5 selective inhibitor, on cognitive function in J20 mice. With administration of Tadalafil for just ten weeks, researchers noted significant improvement in performance on the MWM test when compared to controls. Both Tadalafil and Sildenafil showed a decrease in the phosphorylation of Tau proteins in the mouse hippocampus. These proteins are known to be defective and fail to stabilize microtubules in AD [292]. Moreover, the PDE5 inhibitor UK-343664 was shown to reverse involuntary abnormal movements in a levodopa-induced dyskinesia animal mode, demonstrating the potential benefit of PDE5 inhibition in an experimental paradigm of PD [293].

### 5.6. PDE7

A study by Paterniti et al. revealed the anti-inflammatory effect of PDE7 inhibition after SCI. Two PDE7 inhibitors (S14 and VP1.15), which have been shown to increase intracellular cyclic AMP in vitro in neurons and macrophages, were assessed in vivo after a clip compression SCI in the mouse. When administered intraperitoneally (i.p.), S14 and VP1.15 decreased inflammation and secondary injury in the spinal cord as well as the amount of neutrophil aggregation, inducible nitric-oxide synthase, TNF-α and IL-1β and cyclooxygenase-2 expression, and apoptosis [294]. Medina-Rodriguez et al. (2013) showed that PDE7 inhibition could enhance oligodendrocyte precursor (OPC) survival and accelerate their differentiation in vitro. Treatment with PDE7 inhibitors TC3.6 and VP1.15 also showed promising results for remyelination [295]. Furthermore, Gonzalez-Garcia et al. reported the effect of Rolipram and TC3.6 on experimental autoimmune encephalomyelitis (EAE). While only Rolipram induced an increase in IL-10 and IL-27, both agents decreased IL-17 levels, elevated levels of the T-cell marker Foxp3, inhibited CNS immune cell infiltration and prevented EAE [296]. PDE7 inhibition has been also shown to be effective in treating stroke [297]. Morales-Garcia et al. demonstrated the effectiveness of PDE7 inhibition in Parkinson disease (PD). Inhibiting PDE7 by the heterocyclic small molecule S14 inhibited neuronal death of nigrostriatal dopaminergic neurons, antagonized the activation of microglial cells, and enhanced motor function in a rat PD model [298]. PDE7 inhibition also enhanced cognitive function in an AD mouse model. After 4 weeks of treatment with S14, mice showed a reduction in behavioral deficiencies and pathological markers for AD, suggesting the application of PDE inhibitors, in particular S14, as a prophylactic and therapeutic strategy for AD [299]. Morales-Garcia et al. examined the effectiveness of the combined inhibition of PDE7 and the protein kinase glycogen synthase kinase-3 (GSK-3) in neurological disorders, demonstrating that PDE7 inhibitors acted indirectly to inhibit GSK-3 by elevating cyclic AMP levels and enhancing PKA activation, and when used in combination with GSK-3 inhibitors, led to abrogation of TNF-α and COX-2 following lipopolysaccharide (LPS) challenge in primary astrocyte cultures and reversed LPS-induced glial activation in vivo [300]. Although not confirmed with follow-up studies, a genome wide association study by De Jager et al. investigating possible genetic susceptibility to age-related cognitive decline discovered the rs10808746 allele, found within an intron of PDE7A, to be associated with rapid cognitive decline [301].

### 5.7. PDE9

Huston and colleagues first demonstrated how inhibition of PDE9 could improve cognitive function as well as synaptic plasticity in both rats and mice. With the systematic administration of the PDE9 inhibitor, PF-04447943, they observed an enhanced cognitive level as assessed by various cognitive tests, including novel object recognition [302]. PF-04447943 also prevented memory dysfunction and hippocampal dendritic spine loss in a 4–5-month-old AD mice model. Ultimately, PF-04447943 was shown to elevate central cyclic GMP levels in the brain and CSF. The encouraging preclinical pharmacokinetic studies resulted in the translation of this compound to Phase I clinical trials where it was found to be well tolerated in human volunteers. [303]. Similarly, a study of the PDE9A inhibitor BAY 73-6691 produced encouraging results. When compared to controls, BAY 73-6691 disinhibited long-term potentiation originally impaired by Aβ42 oligomers and improved memory performance in the APP transgenic tg2576 AD mouse model. Like PF-04447943, BAY 73-6691 was found to enhance cyclic GMP levels in the CNS, particularly the hippocampus [304].

### 5.8. PDE10

PDE10A also regulates synaptic plasticity and responses to cortical stimulation [305,306]. Inhibiting PDE10A was shown to have a positive regulatory effect on basal ganglia function, making it a potential target for the treatment of psychosis, Parkinsonism and Schizophrenia [32,303,307,308]. PDE10 inhibition by Papaverine improved spatial and object recognition memory and significantly increased pGluA1 and pCREB levels in the hippocampus when studied in R6/1 mice, suggesting the utilization of PDE10 inhibitors as a therapeutic strategy for the treatment of HD [309]. A study by Giampa et al. revealed that selective inhibition of PDE10A by the drug TP-10 decreased and postponed the hind paw clasping response during tail suspension, improved open field locomotor function, and prolonged time to loss of righting reflex in the R6/2 HD model. Their results also showed significant changes in brain pathology, such as decreased activation of microglia and the development of neuronal nuclear inclusions as well as increased neuronal survival and CREB and BDNF phosphorylation in the cortex and striatum, suggesting the use of PDE10A inhibition as an effective strategy to delay HD progression [310]. PDE10A inhibition is currently in Phase II clinical trials where two doses of the selective and potent PDE10A inhibitor, PF-0254920, is being evaluated for safety, tolerability and brain function in patients with HD [31]. Fittingly, the PDE10A inhibitor Papaverine impaired dopamine D2 receptor signaling while potentiating dopamine D1 receptor signaling through activation of cyclic AMP signaling in striatonigral and striatopallidal neurons. [311].

## 6. Limitations of Currently Available PDE Inhibitors for Therapeutic Use in the CNS

### 6.1. Synthesis

In synthesizing subtype-selective PDE4 inhibitors, a major hindrance has been the structurally conserved catalytic domain present among PDE gene products and isoforms [139,312,313]. A possible way to bypass this issue is to employ allosteric modulators as a means to therapeutically target the different subtypes of these enzymes. Another avenue may include targeting the N-terminal region of the enzyme for inhibition as it contains phosphorylation sites or protein-binding sequences that if blocked would indirectly antagonize the activity of PDE4 [139,313]. The structural and spatial diversity of PDEs make them a sound target for drug development, yet similarities within the conserved domains of PDEs continue to make developing specific and potent PDE inhibitors a challenge. Regardless, UCRs have a tremendous regulatory effect on the PDE4 catalytic region and so appear to be a promising target for catalytic activity modulation. Furthermore, PDE expression in the CNS is incredibly diverse. Thus, inhibition as a modality for the treatment of psychiatric, neurodegenerative, and CNS injury represents an attractive target to possibly precisely modulate neuronal activity if more selective inhibitors can be developed.

### 6.2. Side Effects vs. Potency

For a PDE compound to be considered a “specific” inhibitor of a PDE gene product, it would be expected to exhibit a 50- to 100-fold greater potency for that one PDE over the others. However, occasionally this definition is incomplete as in the case of Sildenafil and Vardenafil, both of which are described as PDE5-specific but also inhibit PDE6 with significant potency [314,315]. This partial inhibition of multiple PDEs at once often becomes a significant issue in the physiological and clinical settings [316,317]. High specificity with a PDE inhibitor is desirable because specificity is typically inversely related to the minimum dosage required for the desired beneficial effect as well as the frequency and severity of side effects [318]. However, the clinical advancement of PDE inhibitors in general has been most hampered by their poor specificity and by extension, their potent side effects. Development of PDE4 inhibitors has been limited, in particular, by a potent emetic side effect profile. When administered intravenously to dogs, Rolipram demonstrated a significant emetic side effect profile accompanied by anxiety and bronchodilatory potency. While intragastric administration significantly minimized the cardiac and respiratory side effects associated with Rolipram, it also minimized the drug’s central nervous system penetrance [319]. Roflumilast and Apremilast, which have now been approved for COPD and psoriatic arthritis, respectively, are recommended at doses which inhibit PDE activity by only 50% since any further increases were observed to induce nausea and emesis [320]. Roflumilast IC50 values for PDE4B are comparable with PDE4D [321]. Although emesis caused by PDE4 inhibition appears dependent on the noradrenergic system, inhibition of PDE4D expression in the area postrema has been particularly implicated [322]. Recently, drug effects on the inner ear, specifically with respect to inner ear fluid homeostasis, have been explored as an alternative etiology for emesis. For example, endolymphatic hydrops, a disorder of the vestibular system, might explain the side effect profile of the drugs. Endolymphatic hydrops has been implicated in neurologic illnesses such as Meniere’s disease, which often present with intractable vertigo and concomitant nausea with emesis, as well as tinnitus and hearing loss. Degerman et al. utilized 9.4T in vivo MRI to observe the effect of Cilostamide, Rolipram, and Sildenafil administrated via mini-osmotic pumps on mouse inner ear fluid balance and found that each of them in turn resulted in endolymphatic hydrops [323]. In addition to emesis, during clinical trials of Roflumilast, serious side effects occurring frequently included diarrhea, atrial fibrillation, and acute renal failure [324]. Methods of eliminating the side effect profile of these compounds without a parallel compromise in potency is thus of particular interest. Simple modifications to regimen or compound synthesis targeting the negative side effect profile associated with PDE4 inhibitors might also be of great clinical import. Structural changes during compound synthesis to enhance specificity might achieve this goal. For example, the PDE4 inhibitors Chlorbipram [325] and GSK356278 [267] were demonstrated to show equal or significantly enhanced brain penetrance when compared to Rolipram, respectively, using models of anti-anxiety or anti-depression, without the induction of emesis. Effectiveness of these inhibitors in the area of neuroregeneration has not yet been tested. GSK356278 was evaluated in pre-clinical and Phase I clinical trials to establish safety and tolerability (single dose, repeat dose, multi dose) [267]. In these Phase I studies, a 14 mg dose of GSK356278 [326] was shown to enter the human brain and occupy nearly 48% of PDE4 enzymes that led to nearly a 11% decrease in the volume of distribution of radio labelled Rolipram. In this study 3/8 enrolled patients reported nausea but none withdrew themselves from the study. Similarly, in another Phase I study [327] looking at the safety profile of varying doses of GSK356278, 4 out of 12 recruited individuals reported nausea as opposed to one out of nine treated with placebo. However, none withdrew themselves from the study. Other clinical studies are supporting that carefully designed dose escalation can circumvent the efficacy-tolerability hurdle for most of the agents in the PDE drug pipeline.

In ferrets, the emetic effects associated with PDE4 inhibitors were attenuated by an adjuvant therapy with 5-HT3 and NK1 receptor antagonists [322,328]. Further studies in human are required to establish a clinically adaptable protocol. Another way of addressing the specificity and side effect profile of PDE4-specific drugs is by developing PDE4 subtype-specific inhibitors [329]. Though challenging due to highly shared catalytic domains, unique regulatory domains of the PDE4s that have a unique first exon could enable structural studies to find novel pharmacological agents (called negative allosteric modulators; NAM) that can bind to conserved pockets in PDE4s in a subtype-specific manner [329]. One such NAM, BPN14770, which targets PDE4D, is currently under a Phase I study for estimating safety and tolerability of single and multiple ascending doses [330]. Recently, a computational study utilized comparative molecular field analysis (CoMFA) and molecular docking to explore the key structural requirements of ligands for PDE4B selectivity and affinity. The study concluded that the ligand docking method can offer superior insights as compared to structure-based drug design and suggested key changes to the chemical structure of prevalent ligands to enable them to putatively be selective for PDE4B over PDE4D [331]. These two studies together have rejuvenated PDE4-based drug development and paved the way for plausible therapies that might be able to harvest proven advantages of enhancing cyclic AMP signaling in various neurological disorders.

Alternatively, another projected side effect that could arise with cyclic AMP-enhancing therapies after CNS damage is aggravated neuropathic pain. Neurotrauma is commonly associated with neuropathic pain and elevated cyclic AMP has indeed been shown to contribute to mechanical hyperalgesia and allodynia, for example by injection of capsaicin [332]. Research has demonstrated that activation of the spinal cord ERK/CREB pathway aggravates constrictive injury-induced neuropathic pain in rats [333]. Although acute CREB injection intrathecally improves tactile allodynia caused by partial sciatic nerve ligation [334], it does so through the stimulation of nerve sprouting, which can in turn promote chronic neuropathic pain. However, a reconditioning nerve lesion can entirely inhibit allodynia associated with a subsequent partial lesion of peripheral nerves [335]. Therefore the relationship between cyclic AMP and neuropathic pain or tactile allodynia is complicated and requires further investigation.

## 7. Clinical Application of PDE Inhibitors in Non-CNS Conditions

Early generation PDE4 drugs lacked specificity. First generation PDE4 inhibitors, including Lirimilast, Filaminast, Tofimilast, and Piclamilast among many others, were all discontinued. Given the lack of specificity, the drug efficacies were very poor at low doses while at high doses the treatment benefits did not outweigh adverse side effect profiles, thus limiting their clinical utility [336]. It seems that great clinical progress was achieved with the second generation, oral, non-selective PDE4 inhibitors, Cilomilast and Roflumilast, for the treatment of COPD [337]. A 2011 Cochrane Library review identified 23 placebo-controlled trials, including both published and unpublished work that included the use of Cilomilast and Roflumilast [338]. The net effect of those trials yielded an average of six month trial duration, totaling 15,668 COPD patients with a wide array of COPD severity [338]. In the majority of cases, while Cilomilast and Roflumilast did show positive results when administered to patients with COPD, the side effect profiles of the PDE4 inhibitors remained [337,339,340,341]. Cilomilast was rejected by the FDA in September 2003 due to the side effect profile of the drug. However, in October 2003, the FDA temporarily approved Cilomilast. The final approval will depend on the outcome of ongoing studies of efficacy and safety [337]. Similarly, in April 2010, the FDA rejected Roflumilast because of the side effect profile. When the European equivalent, the European Medicines Agency, approved Roflumilast later that year, the FDA reconsidered Roflumilast at least for the exacerbation of COPD [342]. Now, Roflumilast (Daxas^®^, Takeda GmbH, Orangeburg, Germany) is the first approved PDE4 inhibitor for COPD treatment on the market [343]. The more recent generation of PDE4 specific inhibitors includes Ogleminlast and IPL512602 (more commonly known as HT-0712). Oglemilast (GRC 3886) did not attain the primary endpoint in a Phase IIb study on asthma patients and as a result research was discontinued by Glenmark Pharmaceuticals.

Alternatives to further pharmacological development have also been thoroughly researched. Burgin et al. demonstrated the value of allosteric modulation by highlighting the differences in upstream conserved regions of PDE4 isoforms [344]. Exploitation of a variation of the amino acid residue at position 196 in the UCR2 among certain isoforms of PDE4 led to creation of a number of PDE4D-selective inhibitors, including PMNPQ, D159404, and D159153 [345]. As a follow up to that work, Fox et al. demonstrated the value of a downstream C-terminal helix (CR3) that can adopt slightly different orientations across the active site after a reciprocal exchange of a single amino acid to convert the selectivity profile between the PDE4B and 4D long isoforms [346]. Separately, utilizing molecular targeting for selective inhibition of PDE4 has also been considered to be an approach with great potential. Such an approach might utilize targeted gene knockout, interference RNA (siRNA), or direct disruption of intracellular pathways, for example [347,348,349,350,351,352,353]. Alternatively, it was determined that administration of Roflumilast with a cyclooxygenase-2 inhibitor (NSAID) could inhibit the majority of the gastrointestinal side effects in rats without compromising efficacy. In rats, co-administration of diclofenac or lumiracoxib has been shown to significantly diminish Roflumilast-mediated spleen and total body weight loss, Roflumilast-mediated diarrhea, and increased secretion of harderian glands [354].

## 8. Conclusions

More than five decades have passed since the discovery of cyclic AMP and its inhibitor family, the cyclic nucleotide phosphodiesterases. PDEs can now be divided into three groups based on their catalytic selectivity to cyclic nucleotides and further subdivided into multiple isoforms and spliced forms. Despite our extensive understanding of PDE4 genetics, the characterization of more than 37 crystal structures of just the PDE4 family alone, as well as details of their expression patterns in various neuropsychiatric disorders and CNS injury paradigms, we have barely scratched the surface of the cyclic AMP signaling landscape for therapeutic application. This notion is supported by newer understanding of cyclic AMP signaling, e.g., Epac-dependent or -independent signaling, divergent cross activities with PKA and the role of regulatory PKA subunits in cellular signaling among others. Even though initial studies demonstrating that cyclic AMP plays an important role in neural plasticity and regeneration and overcoming myelin inhibition are now over a decade old, we have not been able to harvest the fruits of the discovery towards a clinical therapy due to limitations of available PDE inhibitors. The potential of PDEs for various neurological disorders is huge given a wide spread expression, defined ligand and available structures for drug design and docking experiments. Given the broad family structure, we have to understand the relevance of various PDE subtypes in the context of neurological disorders and neurotrauma to better target our efforts for a relevant therapy. This includes developing tools to effectively label PDE subtypes at the RNA and protein level, to track PDE subtypes for visualization purposes and develop highly sensitive yet high-throughput amenable quantification and enzyme activity measures. Another arena to explore would be non-invasive PDE distribution measurement using PET based ligands, this would help to note dynamic changes or task-based changes in PDE activity in neurological disorders and trauma. This type of approach will also enable the move from a static picture of the CNS towards the time scales that engage the cyclic AMP-PDE network.

Preserved importance of PDEs in neurological disorders is exemplified by 206 open studies in 13 different countries around the world registered on the NIH clinical trials website [355] that are currently recruiting. Around 85 studies are investigating some form of neurological disorder ranging from the application of FDA-approved PDE drugs in different neurological scenarios to brand new Phase I trials to determine safety and tolerability of PDEs in AD, stroke and HD. This trend is only expected to improve as soon as newer data regarding PDE4 subtype specific drugs and their generation protocols become available.

## Figures and Tables

**Table 1:** Lead use of PDE inhibitors in Phase I–III clinical trials.

Disease Name	Intervention	Target	Trial Phase	Trial Number
Traumatic brain injury	Sildenafil	PDE5	-	NCT02990078
Alzheimer’s disease	Cilostazol	PDE3	2	NCT02491268
	Roflumilast	PDE4	-	NCT02835716
	BPN14770	PDE4D	1	NCT02840279
	BPN14770	PDE4D	1	NCT02648672
Stroke	Cilostazol	PDE3	3	NCT02481323
	Cilostazol	PDE3	3	NCT01995370
	Tadalafil	PDE5	2	NCT02801032
	Sildenafil	PDE5	1	NCT02628847
Amyotrophic lateral sclerosis	Ibudilast (MN-166)	PDE4	1/2	NCT02714036
Multiple sclerosis	Ibudilast (MN-166)	PDE4	2	NCT01982942
Autonomic nervous system failure and supine hypotension	Sildenafil	PDE5	1/2	NCT00223717
Neonatal encephalopathy	Sildenafil	PDE5	1	NCT02812433
Cerebral vasospasm following sub arachnoid hemorrhage	Milrinone	PDE3	2	NCT02712788
